# Inhibitory Effect of α1 Receptor Antagonists on Paclitaxel-Induced Peripheral Neuropathy in a Rodent Model and Clinical Database

**DOI:** 10.3390/toxics10110669

**Published:** 2022-11-06

**Authors:** Kohei Mori, Takehiro Kawashiri, Keisuke Mine, Mizuki Inoue, Hibiki Kudamatsu, Mayako Uchida, Nobuaki Egashira, Daisuke Kobayashi, Takao Shimazoe

**Affiliations:** 1 Department of Clinical Pharmacy and Pharmaceutical Care, Graduate School of Pharmaceutical Sciences, Kyushu University, Fukuoka 812-8582, Japan; 2 Department of Education and Research Center for Pharmacy Practice, Faculty of Pharmaceutical Sciences, Doshisha Women’s College of Liberal Arts, Kyotanabe 602-0893, Japan; 3 Department of Pharmacy, Kyushu University Hospital, Fukuoka 812-8582, Japan

**Keywords:** paclitaxel, peripheral neuropathy, doxazosin, tamsulosin, large adverse event reporting database

## Abstract

The anticancer drug, paclitaxel, is widely used for ovarian, breast, non-small cell lung, and gastric cancers; however, it induces peripheral neuropathy as a side effect. There is insufficient evidence-based prophylaxis, and new prophylaxis and treatment methods are required. We examined the effect of α1-receptor antagonists on paclitaxel-induced peripheral neuropathy using Sprague-Dawley rats and a large adverse event database. The repeated administration of doxazosin or tamsulosin significantly reduced the response threshold to paclitaxel administration in animal models. In the sciatic nerve tissue, axonal degeneration and myelopathy were significantly suppressed. Furthermore, an analysis of the Food and Drug Administration Adverse Event Reporting System (FAERS) database suggested that the group using α1 inhibitors showed a lower reporting rate for paclitaxel-related peripheral neuropathy than the group that did not use these inhibitors (odds ratio (95% confidence interval): tamsulosin 0.21 (0.08–0.56), *p* < 0.01, doxazosin 0.41 (0.10–1.65), *p* = 0.195; any α1 receptor antagonist 0.54 (0.38–0.76), *p* < 0.01). Thus, doxazosin and tamsulosin may inhibit the development of paclitaxel-induced peripheral neuropathy by suppressing neurodegeneration, particularly axonal degeneration and myelopathy.

## 1. Introduction

Paclitaxel and albumin-suspended paclitaxel are widely used for the treatment of ovarian [1,2], breast [3,4,5], non-small cell lung [6,7], stomach [8,9], uterine [10], and pancreatic [11] cancers; it is known to induce peripheral neuropathy, including numbness, dysesthesia, and pain in the limbs, as a side effect [12]. When severe peripheral neuropathy develops, the treatment must be discontinued or the drug must be changed. Peripheral neuropathy is an obstacle in the continuation of cancer treatment.

The American Society of Clinical Oncology’s (ASCO) “Guidelines for the Management of Peripheral Neuropathy Caused by Anticancer Drugs,” revised in 2020 [13], among others, does not recommend any drug to prevent peripheral neuropathy caused by anticancer drugs, and the only effective drug for the treatment is duloxetine. It is difficult to say whether sufficient countermeasures have been established. Although the results from basic research studies conducted in domestic and foreign research facilities have been gradually reported, there are only a few reports on drugs that prevent or inhibit the root cause of peripheral neuropathy [14], and none have been applied in routine medical care. Therefore, the development of new treatment and prevention methods is desired.

It has been reported that sympathetic signaling mediated by noradrenaline (NAd) and adrenergic α1 receptors is involved in the development and maintenance of neuropathic pain in rat models of sciatic nerve strangulation and diabetic neuropathy [15,16,17,18,19,20]. It is possible that sympathetic signaling is also involved in the development of neuropathic pain induced by anticancer drugs.

On the other hand, doxazosin and tamsulosin are frequently used for dysuria associated with hypertension and benign prostatic hypertrophy, respectively, owing to their α1 receptor-blocking effects. α1 receptor antagonists are effective against diabetic neuropathy [18]. Doxazosin and tamsulosin show neuroprotective effects when used for the treatment of neurodegenerative diseases such as Alzheimer’s disease [21,22]; hence, they can be expected to show efficacy against peripheral neuropathy caused by anticancer drugs.

In this study, we investigated the inhibitory effects of α1 receptor antagonists on paclitaxel-induced peripheral neuropathy (PIPN) by integrating real-world databases and basic pharmacological research.

## 2. Materials and Methods

### 2.1. Drugs

The paclitaxel was purchased from FUJIFILM Wako Pure Chemical Corporation (Osaka, Japan) and was dissolved in 50% ethanol and 50% Kolliphor EL (Sigma-Aldrich Co., St. Louis, MO, USA). The doxazosin mesylate and tamsulosin hydrochloride were purchased from Tokyo Chemical Industry Co., Ltd. (Tokyo, Japan) and were dissolved in 0.5% methylcellulose.

### 2.2. Animals

Male Sprague-Dawley rats (Japan SLC, Shizuoka, Japan) weighing 200–250 g were used for the PIPN effect study and sciatic nerve tissue evaluation. The rats were kept under constant temperature, constant humidity, and a 12 h light–dark cycle (light period 7:00–19:00). Solid feed and water were provided ad libitum. The animal experiments were conducted in accordance with the Kyushu University Animal Experiment Regulations, related laws and regulations, and the ARRIVE guidelines, and were approved by the Kyushu University Animal Experiment Committee.

### 2.3. Analysis of the Effects of Repeated Administration of Alpha 1 Receptor Antagonists on the Development of Peripheral Neuropathy

#### 2.3.1. Drug Administration and Experimental Schedule

Paclitaxel (6 mg/kg) was administered intraperitoneally once a week for 4 consecutive weeks. Doxazosin (30 mg/kg) and tamsulosin (0.4 mg/kg) were administered orally 5 times per week. Concerning the concentration settings of these drugs, we set them with reference to previous reports showing the anti-inflammatory and antioxidant effects, since neuroinflammation and oxidative stress have been reported to be the mechanisms for the development of paclitaxel-induced peripheral neuropathy [23,24]. The body weight was measured every week, and a behavioral study was performed using the von Frey test. In addition, a neurohistological study was conducted using toluidine blue staining of the sciatic nerve.

#### 2.3.2. Von Frey Test

The von Frey test was used as an indicator of mechanical allodynia. The rats were placed on a wire mesh 30 min prior to the test and allowed to acclimate sufficiently. Then, von Frey filaments (Aesthesio^®^; DanMic Global, LLC, San Jose, CA, USA) were measured under the wire mesh at the plantar portion of the right and left hindlimbs for 6 s each time. The up–down method was used for the measurement, and the filament intensity at which the rats showed pain-related responses was recorded as the escape response threshold.

#### 2.3.3. Toluidine Blue Staining

Neuroaxonal degeneration was evaluated by histological assessment. The sciatic nerves were harvested from rats 4 weeks after the drug administration, fixed in 0.1 M of phosphate buffer containing 2% glutaraldehyde (pH 7.4, 4 °C) for 4 h, replaced with an 8% sucrose solution, and after Epon embedding, thin sections were stained with toluidine blue. The stained images of the sciatic nerve cross-sections were analyzed using ImageJ version 1.53 (Wayne Rasband, National Institutes of Health, Bethesda, MD, USA). Circularity was used as an index of the neuroaxonal damage. The g-ratio and thickness of the myelin were used as the indices of myelin sheath damage.

#### 2.3.4. Statistical Processing

The data are presented as the mean ± standard error. The JMP14 software (SAS Institute Inc., Cary, NC, USA) was used for the statistical analysis. A one-way ANOVA was performed to compare multiple groups, and the Tukey–Kramer test was used.

### 2.4. Analysis of the Analgesic Effects of a Single Administration of Alpha 1 Receptor Antagonists on Developed Peripheral Neuropathy

#### 2.4.1. Drug Administration and Experimental Schedule

To evaluate the effects of doxazosin and tamsulosin on mechanical allodynia, the rats were treated with paclitaxel (6 mg/kg) intraperitoneally once a week for 4 consecutive weeks and a von Frey test was performed on the rats with a confirmed expression of mechanical allodynia. Doxazosin (30 mg/kg) and tamsulosin (0.4 mg/kg) were suspended in 0.5% methylcellulose and administered orally as a single dose after the development of allodynia due to paclitaxel administration. The tests were performed before and after the drug administration at 30, 60, 90, and 120 min.

#### 2.4.2. Statistical Processing

The data are presented as a mean ± standard error. The JMP14 software was used for the statistical analysis. Comparisons between the two groups were analyzed with a Student’s *t* test. A one-way ANOVA was performed to compare multiple groups, and the Tukey–Kramer test was used.

### 2.5. Evaluation of PIPN Suppression Effects Using a Large Adverse Event Database

#### 2.5.1. Analysis of FAERS Data

To evaluate the effect of the PIPN suppression, data were extracted using CzeekV Pro (Version 5.0.32, Intage Healthcare Corporation, Tokyo, Japan, accessed April 2021) for analyzing the data reported by FAERS from 2004 to 2020. Of the 13,829,818 adverse event reports registered during the period of interest, 71,351 adverse event reports pertaining to paclitaxel usage were included in the study. The α1 receptor antagonists, number of PIPN reports with and without concomitant use of each drug, reporting rates, and reporting odds ratio (ROR) were analyzed. The PIPN was defined as reports pertaining to peripheral neuropathy, peripheral sensory neuropathy, or peripheral sensorimotor neuropathy in patients treated with paclitaxel. The ROR and 95% confidence interval (CI) were calculated using the following formulae:

ROR = n11/n21n12/n22(1)

95% CI = exp[log(ROR) ± 1.96√1/n11 + 1/n12 + 1/n21 + 1/n22](2)

In the above equation, n11 represents patients who used concomitant medications and reported PIPN, n12 represents patients who used concomitant medications and did not report PIPN, n21 represents patients who did not use concomitant medications and reported PIPN, and n22 represents patients who did not use concomitant medications and did not report PIPN.

#### 2.5.2. Statistical Processing

The JMP14 software (SAS Institute Inc., Cary, NC, USA) was used for the statistical analysis. Each data point was tested using the chi-squared test. A Yates’ correction was performed when the number of cases for each item was less than 5. A risk rate of less than 5% (*p* < 0.05) was considered as a significant difference.

## 3. Results

### 3.1. Effects of Repeated Administration of Doxazosin and Tamsulosin on Paclitaxel-Induced Mechanical Allodynia in Rats

**Figure 1:**
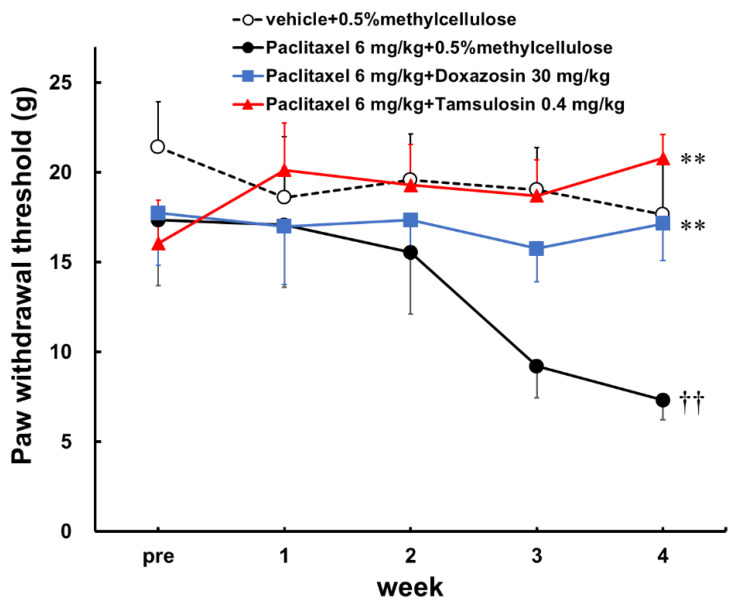
Effects of doxazosin and tamsulosin on paclitaxel-induced mechanical allodynia in SD rats. Paclitaxel (6 mg/kg) was administered intraperitoneally once weekly for 4 weeks. Doxazosin (30 mg/kg) and tamsulosin (0.4 mg/kg) were administered orally 5 times a week for 4 weeks. A von Frey test was performed before the first drug administration (pre) and then once a week. Threshold values were determined to be mean ± S.E.M. (*n* = 6–8). †† *p* < 0.01 vs. vehicle + 0.5% methylcellulose; ** *p* < 0.01 vs. paclitaxel + 0.5% methylcellulose, one-way ANOVA followed by a Tukey–Kramer test.

The repeated administration of paclitaxel (6 mg/kg) caused a significant decrease in the response threshold at the 4th week of treatment (Figure 1; week 4, *p* < 0.01). The repeated administration of tamsulosin (0.4 mg/kg) and doxazosin (30 mg/kg) significantly prevented the paclitaxel from lowering the response threshold (Figure 1; week 4, *p* < 0.01).

### 3.2. Effects of Repeated Administration of Doxazosin and Tamsulosin on Paclitaxel-Induced Neuroaxonal Degeneration in Rat Sciatic Nerves

**Figure 2:**
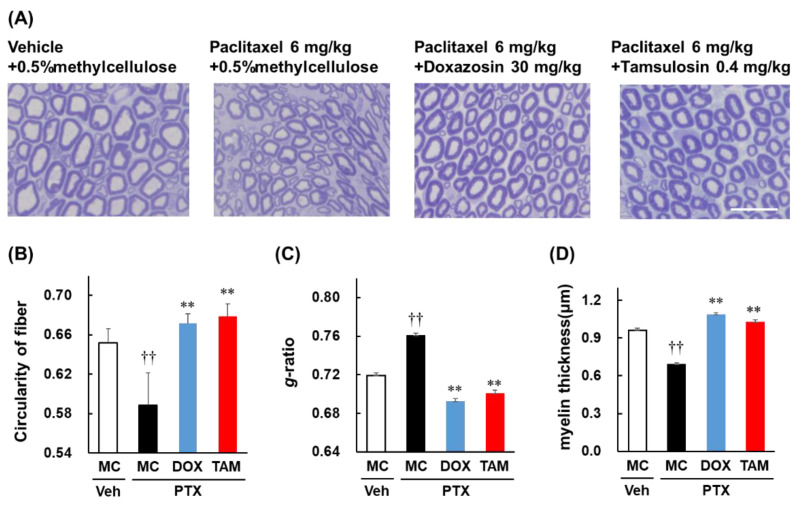
Effects of doxazosin (DOX) and tamsulosin (TAM) on axonal degeneration and myelin sheath damage of the sciatic nerve induced by paclitaxel (PTX) in rats. The sciatic nerve was harvested on day 28 and stained with toluidine blue. Images (shown in **A**) have been magnified 40× (bar  =  25 µm). Fiber circularity (**B**), g-ratio (**C**), and myelin sheath thickness (**D**) were analyzed using ImageJ (version 1.53) software. These results have been presented as mean ± S.E.M. †† *p* < 0.01 vs. vehicle + 0.5% methylcellulose, ** *p* < 0.01 vs. paclitaxel + 0.5% methylcellulose; one-way ANOVA followed by a Tukey–Kramer test.

Figure 2A shows a cross-sectional image of the rat sciatic nerve tissue stained with toluidine blue. The blue-stained area is the myelin sheath and the white area inside it is the axon. Each axon was evaluated for circularity. Significant axonal degeneration was observed in the paclitaxel-only group (*p* < 0.01; Figure 2B), whereas a significant suppression of axonal damage was observed in the groups where paclitaxel was combined with doxazosin or tamsulosin (Figure 2B). The myelin sheath damage was assessed using the g-ratio and thickness of the myelin sheath. Significant myelopathy was observed in the paclitaxel-only group (*p* < 0.01; Figure 2C,D), whereas it was significantly reduced in the groups where the paclitaxel was combined with doxazosin or tamsulosin (*p* < 0.01; Figure 2C,D).

### 3.3. Effects of the Single Administration of Doxazosin and Tamsulosin on Developed Paclitaxel-Induced Mechanical Allodynia in Rat Sciatic Nerves

**Figure 3:**
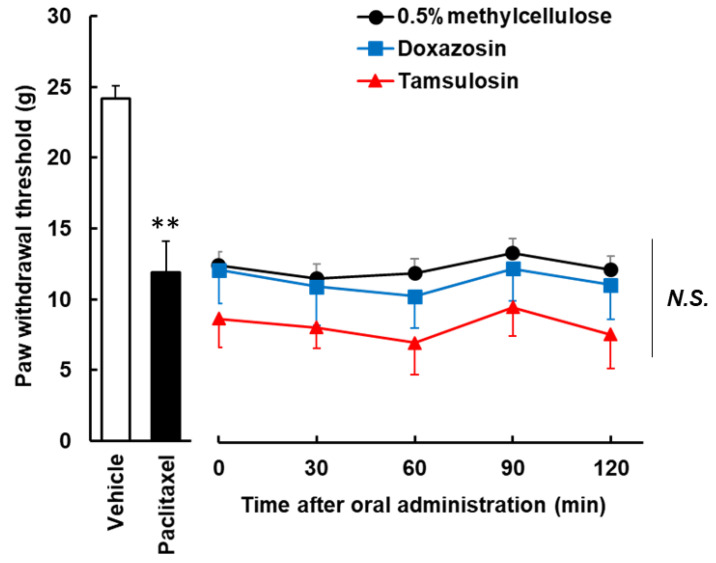
Effects of single administration of doxazosin and tamsulosin on developed paclitaxel-induced mechanical allodynia in the von Frey test. Paclitaxel (6 mg/kg) was administered intraperitoneally once weekly for 4 weeks. Doxazosin (30 mg/kg) and tamsulosin (0.4 mg/kg) were administered orally as a single dose after the development of allodynia due to paclitaxel administration. Threshold values were determined to be mean ± S.E.M. (*n* = 8). ** *p* < 0.01 vs. vehicle, Student’s *t* test.

The doxazosin (30 mg/kg, p.o.) and tamsulosin (0.4 mg/kg, p.o.) did not transiently relieve paclitaxel-induced mechanical allodynia (Figure 3).

### 3.4. Evaluation of PIPN Suppression Using a Large Adverse Event Database

**Figure 4:**
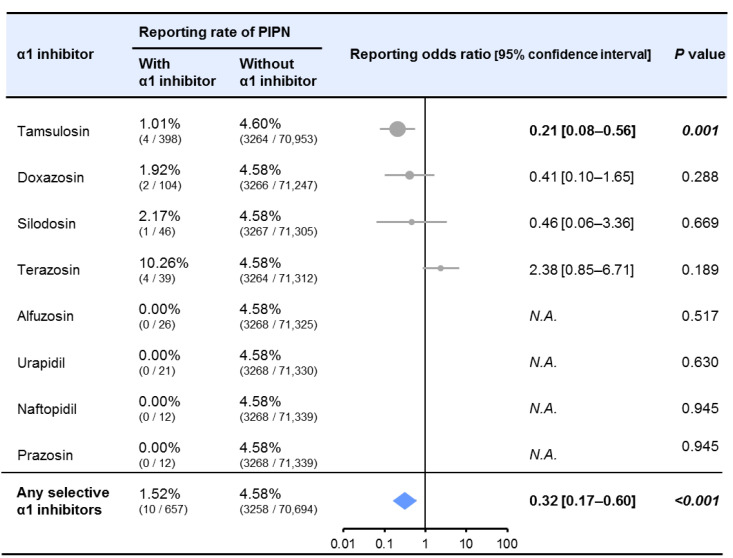
Effects of α1 receptor antagonists on reporting ratio of paclitaxel-induced peripheral neuropathy (PIPN) in Food and Drug Administration Adverse Event Reporting System (FAERS). The reported data were extracted using CzeekV Pro (version 5.0.23, INTAGE Healthcare Inc., Tokyo, Japan, accessed April 2021). A total of 71,351 adverse event reports from patients using paclitaxel were included in this study. PIPN was defined as reports pertaining to peripheral neuropathy, peripheral sensory neuropathy, or peripheral sensorimotor neuropathy in patients using paclitaxel.

Of the 71,351 adverse event reports from patients using paclitaxel in the FAERS database, 3268 (4.58%) were PIPN reports, indicating that the incidence of PIPN was significantly lower when using tamsulosin and may be lower with doxazosin (Figure 4). The reported odds ratio (ROR) for the PIPN were 0.41 (95% confidence interval (CI) 0.10–1.65; *p* = 0.288) for the doxazosin and 0.21 (95% CI 0.08–0.56; *p* = 0.001) for the tamsulosin. Furthermore, the incidence of PIPN was significantly lower in the patients taking any of the concomitant α1-receptor antagonists (ROR (95% CI) = 0.32 (0.17–0.60), *p* < 0.001).

## 4. Discussion

In the Sprague-Dawley rats, the repeated administration of doxazosin or tamsulosin significantly reduced mechanical allodynia. In the sciatic nerve tissue, axonal injury and myelopathy were significantly suppressed. These results suggest that doxazosin and tamsulosin may inhibit PIPN by suppressing neurodegeneration, particularly axonal injury and myelopathy. In the present study, a single dose of doxazosin or tamsulosin had no analgesic effect on the already developed PIPN. Thus, these results suggest that the action of α1-receptor antagonists on PIPN is not a transient analgesic effect.

Paclitaxel is a taxane-based antineoplastic agent used against many types of cancer, including ovarian [1,2], breast [3,4,5], non-small cell lung [6,7], stomach [8,9], uterine [10], and pancreatic [11] cancers. However, paclitaxel causes peripheral neuropathy, including pain and numbness in the limbs and abnormal temperature sensation in many patients, making it difficult to continue the chemotherapy. The guidelines, including “Guidelines for the Management of Peripheral Neuropathy Caused by Anticancer Drugs”, established by ASCO [13], state that no drug is recommended for the prevention of peripheral neuropathy caused by anticancer drugs and that duloxetine is the only effective drug for the treatment of such neuropathy; however, the ability of duloxetine to suppress neuropathic pain caused by taxanes was shown to be weak [25]. Thus, there is currently no adequate evidence-based treatment for PIPN.

Basic research has partially clarified the mechanism by which paclitaxel induces neurodegeneration. Paclitaxel induces axonal damage by inhibiting microtubule depolymerization in the peripheral nerve axons [26]. It also induces myelopathy, which is essentially caused by the axonal damage. In this study, the α1 receptor antagonists, doxazosin and tamsulosin, were shown to significantly inhibit paclitaxel-induced axonal degeneration and myelopathy and to significantly suppress neuropathy. In a previous report, tamsulosin was shown to improve memory [27]. Tamsulosin has been demonstrated to exert neuroprotective effects against intracerebral hemorrhage by inhibiting hippocampal cell apoptosis via a blockade of the α1 adrenergic receptors [28]. Doxazosin has also been reported to exhibit neuroprotective effects by suppressing oxidative stress and inflammatory cytokine production via α1 receptors [29]. Moreover, doxazosin and tamsulosin have also been reported to exhibit anti-inflammatory effects in several animal models [30,31]. Furthermore, paclitaxel induces macrophage infiltration of the rat spinal dorsal root ganglion (DRG) and promotes the expression of inflammatory cytokines such as interleukin-1 (IL-1) and tumor necrosis factor-α (TNF-α) [32,33]. The intrathecal administration of an IL-1 receptor antagonist and plasmid DNA encoding the anti-inflammatory cytokine, interleukin 10, restored paclitaxel-induced mechanical allodynia [34]. Although the mechanism of paclitaxel-induced peripheral neuropathy is not yet fully understood, inflammatory cytokines may be involved in the pathogenesis of this adverse effect. Taken together, these agents may have suppressed the PIPN by inhibiting neuroinflammation. In addition, it has been reported that doxazosin is distributed systemically in rats, including the central nervous system, promptly after administration [35]. Therefore, it seems logical that doxazosin would also be directly involved in neuroprotection.

Although many drugs have been reported to be effective against PIPN in basic research, they have not yet been translated into clinical applications. One way to bridge the gap between basic research and clinical practice is to analyze databases that collect clinical medical information; one such database is the FDA Adverse Events Database for Drugs (FAERS), which contains a large number of drug-related adverse event cases and is, therefore, not only effective as a comprehensive risk management tool but also as a source of clinical information on drug-related adverse events. Recently, this database has been employed in drug safety evaluation and drug discovery and development research [36,37,38,39,40].

No side effects, including staggering due to hypotension, were observed with the use of the alpha 1 receptor antagonists during the experiment. Tamsulosin and doxazosin are drugs already in clinical use for dysuria and hypertension, respectively, and have few side effects. Hypotension is the most major side effect of both drugs. Therefore, doxazosin and tamsulosin may represent a new prophylactic option for PIPN in hypertensive patients. Moreover, tamsulosin is usually used for dysuria associated with prostate enlargement, and there are extremely few safety data or clinical experiences with women. Additional evidence is needed regarding information on side effects such as hypotension and indications for use in women. Recently, several randomized trials have reported that cryotherapy using cold gloves reduces PIPN [41,42,43,44]. Combining such non-pharmacologic therapy with pharmacologic therapy, including α1 receptor antagonists, may efficiently reduce peripheral neuropathy. A high BMI is a risk factor for taxane-induced peripheral neuropathy [45,46], and is also associated with hypertension [47]. Moreover, women with a high BMI are at an increased risk of postmenopausal breast cancer [48]. Taken together, the use of α1 receptor antagonists to suppress PIPN in patients with a high BMI, breast cancer, and hypertension may be worthwhile and reasonable.

The present study suggests that the incidence of PIPN was significantly reduced by tamsulosin and may be reduced by doxazosin based on the FAERS analysis. The ROR was also significantly decreased by both α1 receptor antagonists. Although the safety of tamsulosin in women has not been established, other α1 receptor antagonists besides tamsulosin can be considered in women, as α1 receptor antagonists were shown to significantly reduce the incidence of PIPN in FAERS. Notably, doxazosin causes hypotension as a side-effect; therefore, doxazosin may be a novel prophylactic option against PIPN in patients with hypertension. In the future, basic research should examine whether selective α1 receptor antagonists other than doxazosin and tamsulosin, nonselective α receptor antagonists, and αβ receptor antagonists have inhibitory effects on PIPN, and it should elucidate the neuroprotective mechanisms on PIPN. In addition, it is necessary to establish evidence for the benefit of α1 receptor antagonists for PIPN in clinical studies.

## 5. Conclusions

In this study, using a combination of basic research using animal models and a large adverse event database, we demonstrated that doxazosin and tamsulosin inhibit the development of PIPN; therefore, doxazosin and tamsulosin may be used as new prophylactic options against PIPN.

## Data Availability

The data that support the findings of this study are available from the corresponding author upon reasonable request.
